# Disseminated *Verruconis gallopava* in a kidney transplant recipient on belatacept, who failed isavuconazole and improved on dual amphotericin B and posaconazole therapy

**DOI:** 10.1016/j.mmcr.2026.100802

**Published:** 2026-06-02

**Authors:** Matthew Cappiello, Matthew Carlin Corpuz Bonus, Sandy Chang, Jonathan Arcobello

**Affiliations:** a Division of Infectious Diseases, Loma Linda University Medical Center, 11234 Anderson St, Loma Linda, CA, 92354, USA; b Department of Clinical Laboratory Science, Loma Linda University Medical Center, 11234 Anderson ST, Loma Linda, CA, 92354, USA

**Keywords:** Phaeohyphomycosis, Verruconis, Disseminated fungal infection, Infection in immunocompromised host, Mold infection

## Abstract

Disseminated phaeohyphomycosis can have severe consequences in immunocompromised hosts, and does not have standardized therapeutic protocols. A 68-year-old man with prior renal transplant presented with nodular pneumonia. Symptoms worsened despite empiric antimicrobials, including isavuconazole for antifungal coverage. After new T2-enhancing lesions were seen on MRI brain along with worsened pulmonary and soft tissue lesions, transbronchial biopsy revealed disseminated *Verruconis gallopava* as the inciting pathogen. Therapy was successfully narrowed to posaconazole based on sensitivities. Future literature should evaluate the most optimal first-line therapy for *Verruconis*, due to the high morbidity and mortality seen in disseminated disease.

## 1 Introduction

Phaeohyphomycosis is a term used to describe a group of invasive fungal infections caused by dematiaceous fungi with approximately 70 genera and 150 different species known to cause human infection [1]. Clinical manifestations may vary from superficial, subcutaneous, or disseminated, and is distinguished from other syndromes caused by dematiaceous fungi [2] such as eumycotic mycetoma or chromoblastomycosis specifically through the presence of pigmented septate hyphae causing tissue invasion. Melanic pigment in these organisms are thought to be a key virulence factor acting as a free radical scavenger that limits phagocytosis [2], along with protease and peptidase enzymes as well other factors conferring antifungal resistance [1,3].

Phaeohyphomycosis affects both immunocompetent and immunocompromised patients. A Transnet database study suggested a 2.6% incidence of phaeohyphomycosis, distributed equally among solid organ and hematopoetic stem cell transplants [4]. While only limited data exists for phaeohyphomycosis epidemiology, these and other dematiaceous mold infections have been isolated from the southeastern continental United States, associated with sewage and animal litter settings as well as extreme weather events [5].

## 2 Case presentation

A 68-year old man with a history of end-stage renal disease with subsequent deceased donor renal transplant nine months prior to admission presented on day 0 with shortness of breath and dry cough for a three-month duration. Review of systems included a pustule on his chest for the last two weeks (Fig. 1). He had no sick contacts, nor any other new travel, dietary, nor zoonotic exposures.

**Fig. 1:**
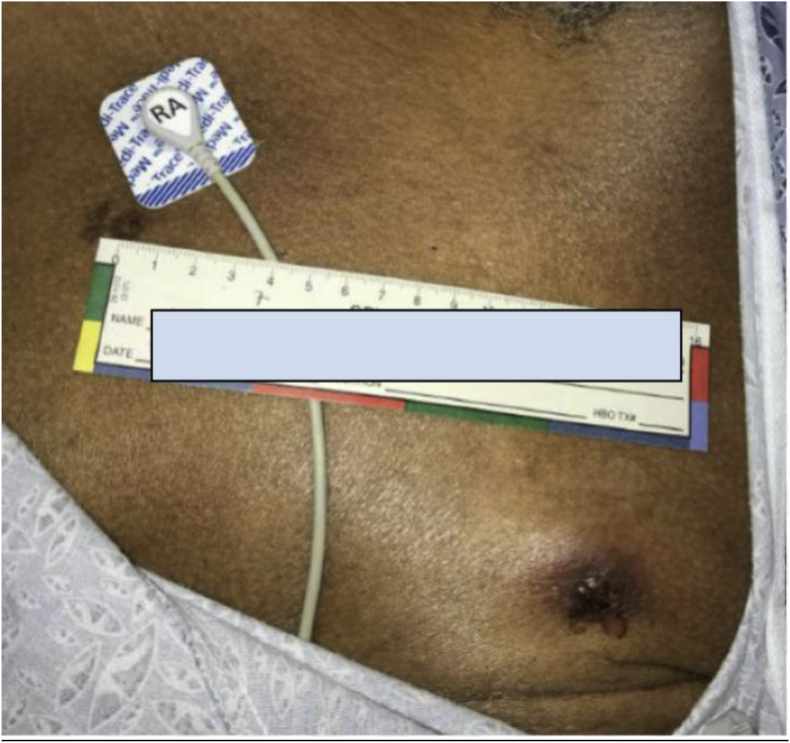
Left-sided chest pustule, present on admission.

Induction consisted of anti-thymocyte globulin followed by maintenance immunosuppression with mycophenolate sodium, tacrolimus, and prednisone. He had delayed graft function, and a renal biopsy two months after transplant showed interstitial fibrosis and tubular atrophy. As there was concern for the possibility of atypical hemolytic uremic syndrome, he was started on intermittent belatacept at five months post-transplant, with the last dose occurring two weeks before admission. In addition, the patient had a previous history of a positive *Coccidioides* IgG titer, and he was maintained on fluconazole prophylaxis 200mg daily after transplantation per institutional protocol.

The patient was afebrile on admission, with vitals stable and otherwise on room air. Exam showed a small open wound on his mid-chest with purulent drainage (Fig. 2). Labs revealed a mild neutrophilic leukocytosis of 10.8 bil/L with 77% neutrophils, but otherwise with unremarkable electrolyte, liver, or kidney function. Computed tomography (CT) angiography of his chest (Fig. 3) displayed a 4.1cm right lower lobe mass-like consolidation, as well as a 1.7cm left upper lobe pulmonary nodule. CT abdomen was unremarkable.

**Fig. 2:**
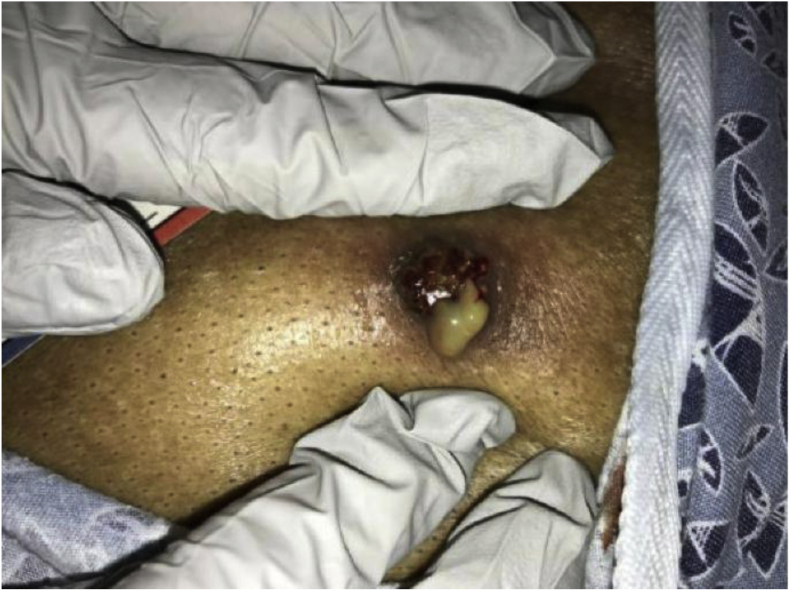
Expression of purulence from left-sided chest pustule.

**Fig. 3:**
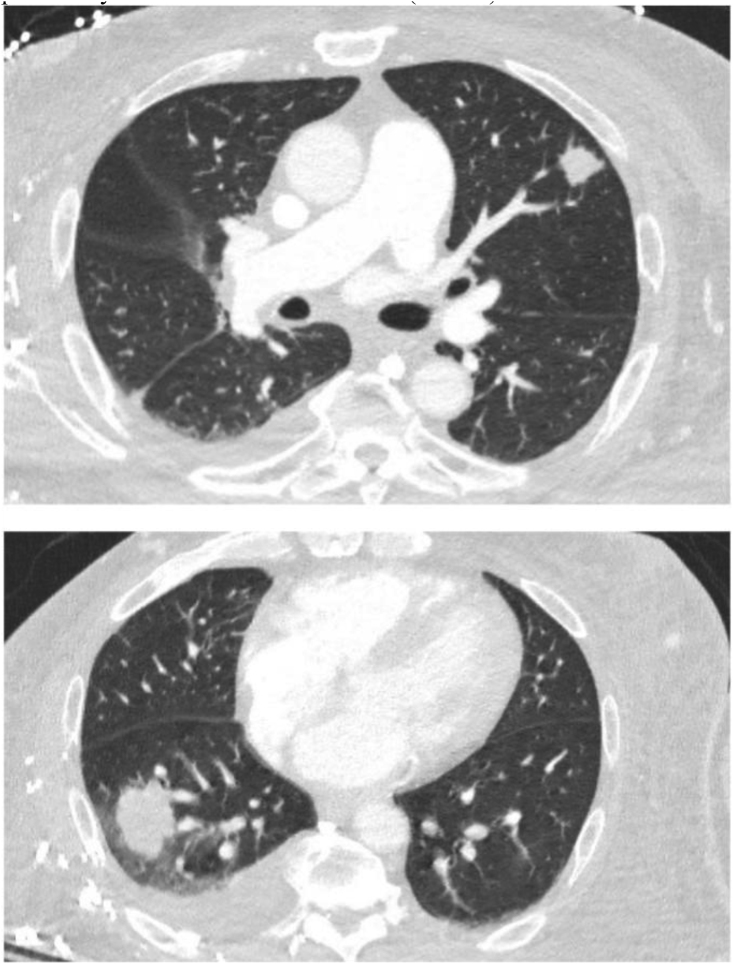
Left upper lobe pulmonary nodule on admission (top), as well as right lower lobe pulmonary consolidation on admission (bottom).

The patient's mycophenolate was held on hospital day 0. He received an incision and drainage of his chest wound on day 2. Transplant Infectious Diseases was consulted, recommending empiric linezolid, meropenem, and isavuconazole loading dose followed by 372mg daily maintenance dose for broad bacterial and fungal coverage with more preferable toxicities and drug interactions while awaiting further workup. Magnetic resonance imaging (MRI) of face and sinus was unremarkable. Serum *Aspergillus*, *Cryptococcus*, *Coccidioides*, and *Histoplasma* serologies were unremarkable, although 1,3-B-D-glucan was highly elevated above the detectable assay threshold at >500 pg/mL. Preliminary wound cultures from the chest grew filamentous fungi within 24 hours (Fig. 8), showing olive-black velvety colonies.

Despite over one week of isavuconazole therapy in the setting of filamentous fungi on cultures, the patient continued to develop signs of worsening infection, with worsening leukocytosis and hypoxia, as well as new delirium and word-finding difficulties without other focal deficits on neurologic exam. Repeat CT chest on day 10 showed increased right lung consolidation as well as multiple new pulmonary nodules, including one in the left upper lobe now showing cavitation (Fig. 4). MRI brain on day 12 showed a T2/FLAIR hyperintense left frontal lobe lesion with hemosiderin staining, suggestive of central nervous system (CNS) involvement. (Fig. 5). Physical exam showed a small nondraining subcutaneous nodule on the patient's buttock, and CT pelvis showed a 5.0cm peripheral enhancing right gluteal fluid collection, in the setting of new right flank pain (Fig. 6). Bronchoalveolar lavage (BAL) fungal culture grew filamentous fungi after 6 days of incubation, but was negative for *Aspergillus* BAL galactomannan and also negative for sendout Mayo Clinic targeted polymerase chain reaction (PCR) validated in BAL fluid for coccidiomycosis, histoplasmosis, blastomycosis, and *Pneumocystis*. BAL bacterial and acid fast cultures were also negative. Pathology of transbronchial biopsy also showed filamentous fungal forms, with background granulomatous inflammation (Fig. 7). Gluteal fluid aspiration also grew filamentous fungi on cultures, within 72 hours of incubation.

**Fig. 4:**
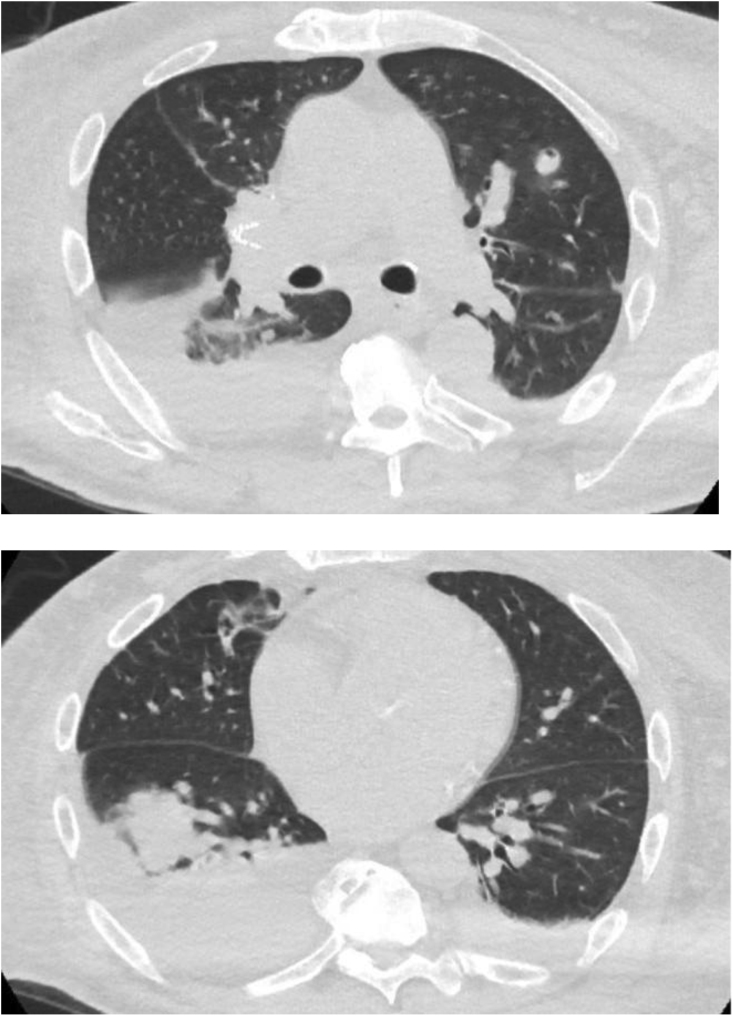
Worsening CT chest after 72 hours of empiric antifungal and antibacterial therapy, including worsening cavitation of left upper lobe nodule (top) as well as worsening right lower lobe consolidation (bottom).

**Fig. 5:**
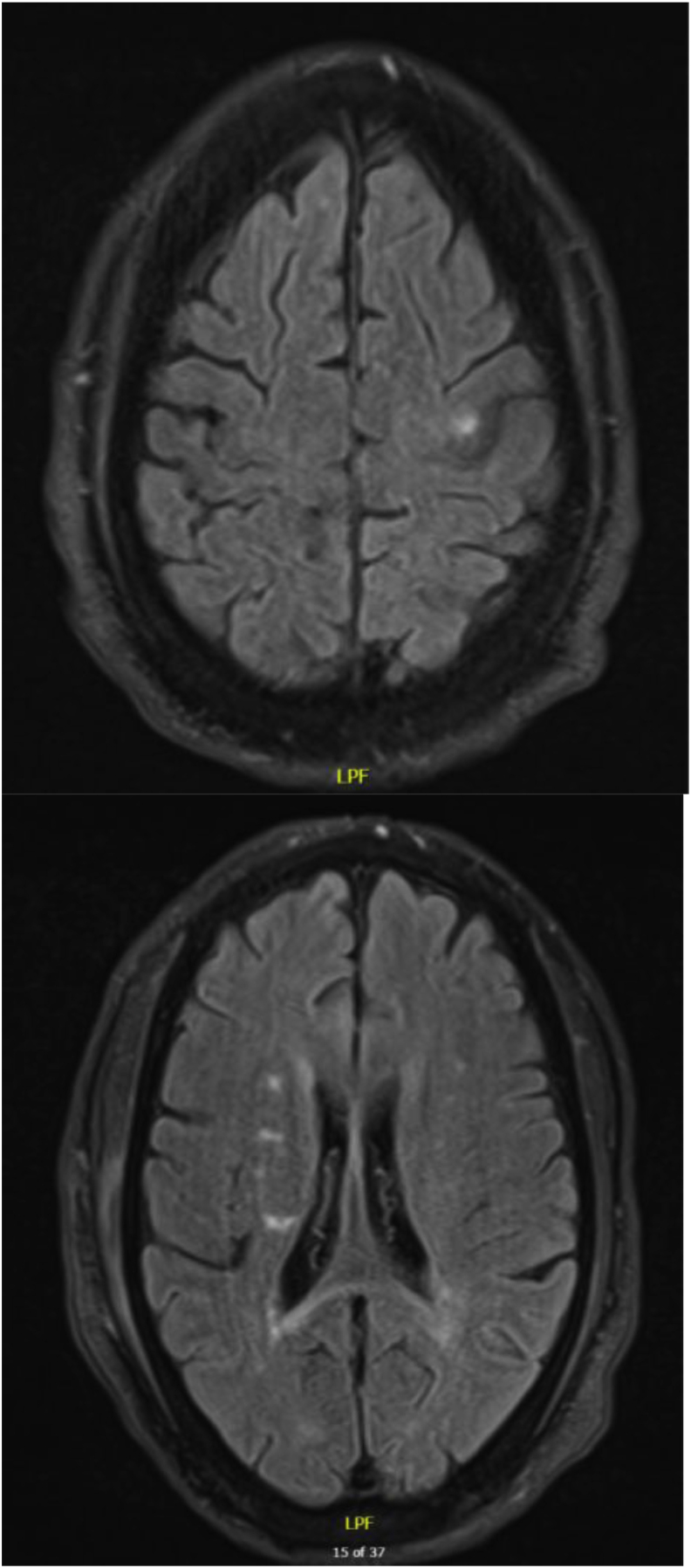
MRI brain, showing T2/FLAIR hyperintense left frontal lobe lesion with hemosiderin staining, suggestive of CNS spread of disseminated infection.

**Fig. 6:**
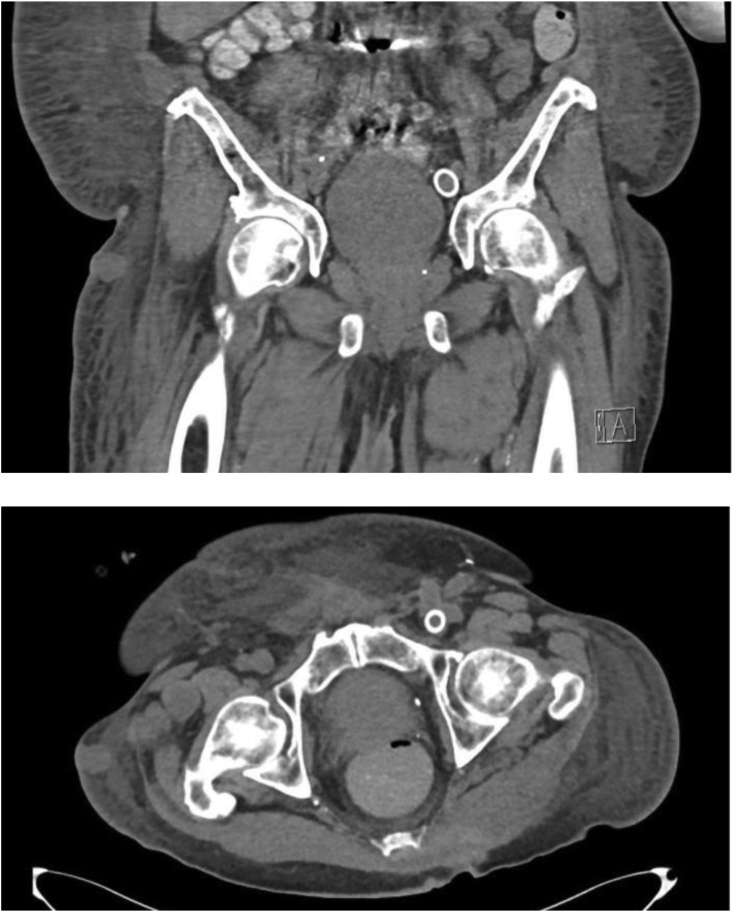
CT pelvis, with a 5.0cm peripheral enhancing right gluteal fluid collection.

**Fig. 7:**
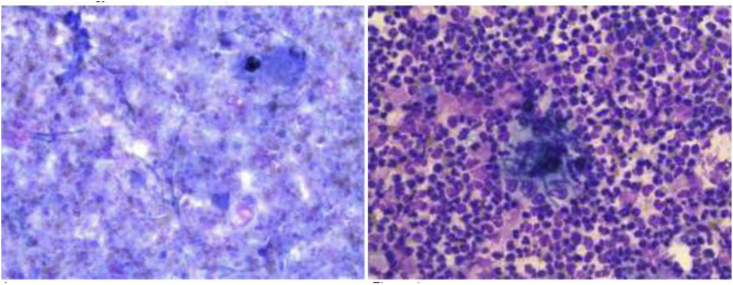
Transbronchial biopsy from bronchoscopy, with pathology showing filamentous fungal forms (left), with background granulomatous inflammation (right).

**Fig. 8:**
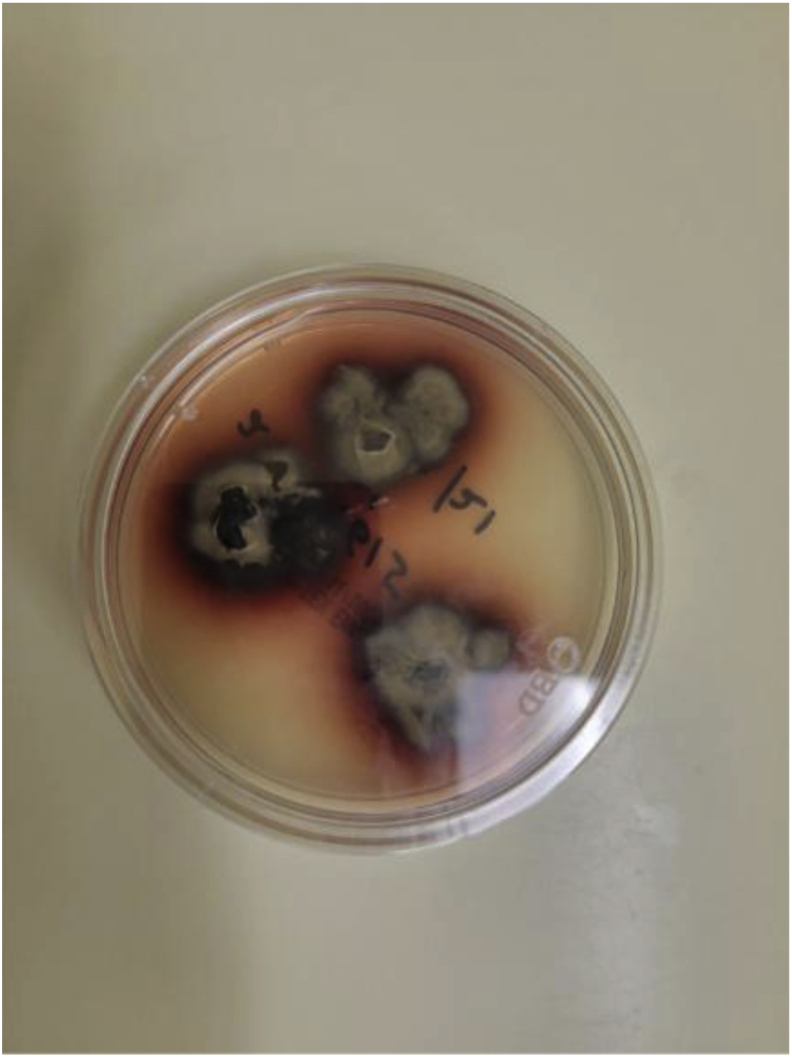
Microbiology lab plate identification, with dematiaceous mold morphology.

Plate identification using Saboraud agar at 30 °C showed dematiaceous mold (Fig. 8), with septate hyphae seen on microscopy (Fig. 9). Species was ultimately identified on all body fluid cultures as *Verruconis gallopava*, based on macroscopic and microscopic characteristics with positive lactophenol cotton blue staining as well as thermophilic features [6]. Subsequent sendout sensitivities to University of Texas Health Sciences San Antonio's lab per CLSI M38-A2 guidelines demonstrated a micafungin minimum inhibitory concentration (MIC) ≤ 0.015 mcg/mL, posaconazole MIC ≤ 0.03, voriconazole MIC of 1, isavuconazole MIC of 8, itraconazole MIC of ≤ 0.03, amphotericin MIC of 0.5, and fluconazole MIC of 64. Serum isavuconazole level was found to be therapeutic at 3.2 mcg/mL.

**Fig. 9:**
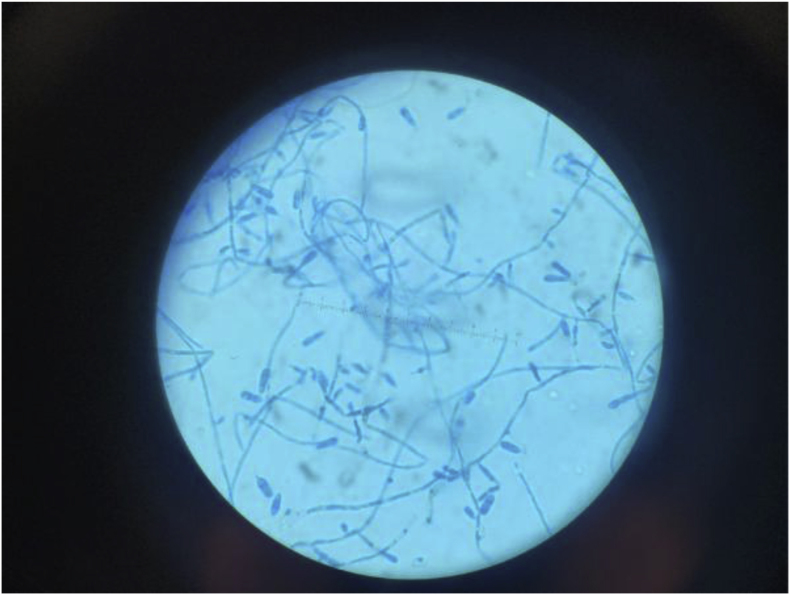
Microbiology lab microscopy, showing septate hyphae.

The patient was ultimately diagnosed on hospital day 12 with disseminated phaeohyphomycosis due to *Verruconis gallopava*, with evidence of pulmonary, CNS, and soft tissue spread. Antifungal therapy was optimized to dual coverage with amphotericin 5mg/kg daily, as well as posaconazole 300mg delayed-release tablets twice daily, upon organism speciation, with isavuconazole discontinued after a total of 10 days of therapy. (Posaconazole twice-daily dosing is frequently utilized to obtain therapeutic levels, but is not precisely according to prescribing instructions and can occasionally prolong the QT interval.) Follow-up MRI brain and CT chest on hospital day 22 showed improvement, corresponding to ten additional days of therapy on this new regimen. Patient developed worsening allograft function and was started on hemodialysis on hospital day 17. His belatacept dose was due on hospital day 21 and this was held in setting of disseminated fungal infection; prednisone and tacrolimus were continued.

The patient was transitioned to posaconazole monotherapy on discharge at hospital day 39, based on fungal sensitivities. Posaconazole level was found to be therapeutic at 2780 ng/mL, within the target treatment trough and below the toxicity ceiling [7] At outpatient transplant infectious diseases clinic two weeks after discharge, he continued to show clinical and radiologic improvement on follow up evaluation, including improved appearance of his original chest wound (Fig. 10). At the time of publication, it was tentatively planned to continue at least a total of twelve-month course of treatment with posaconazole.

**Fig. 10:**
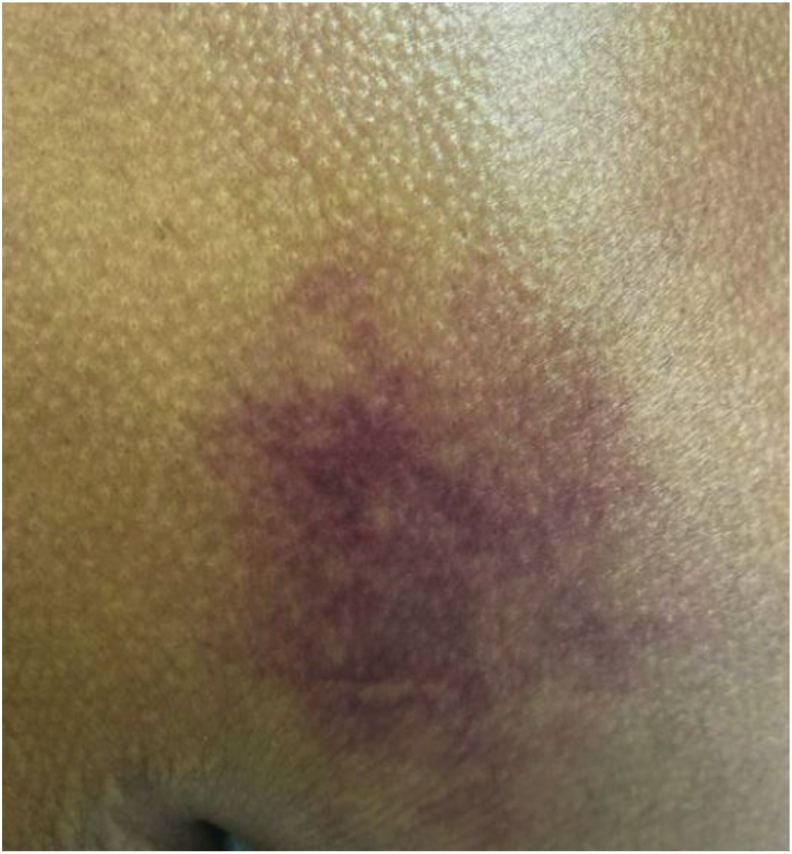
Follow-up photography of L-sided chest wound two weeks after hospitalization, showing significant improvement.

## 3 Discussion

*Verruconis* is a melanized saprophytic mold that has been traditionally isolated from nutritionally poor environments, including thermal springs, soil, decaying vegetation, chicken litter, and even nuclear reactor effluent. Most cases of human infection occur in the southeastern United States [8], which was our patient's original location of residence. Deep infections have also been noted in veterinary species including birds and poultry as well as human pets such as dogs and cats. Colonies are morphologically smooth to suede, with brown diffusible pigment and cylindrical conidiophores [1].

Clinical syndromes of phaeohyphomycosis are manifold, including superficial and deep local infections as well as disseminated disease with pulmonary and CNS manifestations [1]. The most common presentation of *Verruconis* and *Ochroconis* is pulmonary disease, although presentation is highly variable and more commonly occurs in immunocompromised patients, similar to this patient's case. Of note, up to 8-17% of all keratitis in tropical regions is also thought to be due to dematiceous fungal infections such as this condition. Disseminated and CNS disease has high mortality rates, with >50% and >70% mortality respectively [9]. While pre-transplant airway colonization is thought to be less common than other invasive molds such as *Scedosporium* and *Phialomonium* [8], invasive pneumonia can still occur in phaeohyphomycosis, sometimes accompanied by allergic bronchopulmonary disease and/or sinusitis. Of note, our patient had been on belatacept post-transplant. This is one of the first documented cases of belatacept-associated dematiaceous mold infection. Belatacept has been previously associated with invasive fungal infections [10] but not specifically phaeohyphomycosis.

Diagnostics remain challenging for confirmation of phaeohyphomycosis, with no specific serology or PCR available to date. Careful culture and pathology review remains the mainstay of diagnosis [1]. A high clinical index of suspicion is often necessary, including immunocompromised patients such as this patient who had symptoms in multiple organ systems that did not fit a classical clinical pattern. Histopathology shows granulomatous inflammation with multinucleated giant cells, suppurative areas, and dermal fibrosis. Phaeohyphomycotic cysts can demonstrate yeast-like fungal elements with septated hyphae, with additional presence of foreign body splinter from original inoculation of infection. Fontana Masson stain is specific for melanin in these species [9], which is key to distinguish hyalohyphomycosis from phaeohyphomycosis. Histopathologic diagnosis should also include presence or absence of tissue invasion, as well as host reaction to infection. Only one-fourth of specimens demonstrate necrosis, and even less commonly a defined granuloma. Future matrix-assisted laser desorption ionization–time of flight (MALDI-TOF) library enrichment and PCR diagnostics [11] may assist with more rapid diagnosis.

With regards to therapeutics, treatment regimen is often centered in the literature around culture identification of specific organisms as well as locations of infection. As *Verruconis* frequently involves neurotropic CNS and disseminated manifestations, complete excision is recommended in addition to combination antifungal therapy. Although triazoles are normally thought to have excellent activity for phaeohyphomycoses [12], no standardized therapies or interpretive breakpoints exist for most antifungal drugs. The optimal duration of therapy is also unknown. Length is often personalized on a case-by-case basis, based on clinical response. For our patient, we chose an initial duration of 12 months based on available case reports [13,14] with plan to possibly extend based on clinical and radiologic resolution. The European Society of Clinical Microbiology and Infectious Diseases (ESCMID) and European Confederation of Medical Mycology (ECMM) has published joint clinical guidelines for diagnosis and management of systemic phaeohyphomycosis [15] as well as hyalohyphomycosis [16], with species-specific brief reviews of major pathogens.

With regards to *Verruconis*, most antifungal drugs have low MICs in this genus. However, as for other dematiaceous fungi, there are no interpretive MIC breakpoints and no recommendations for the use of MICs to guide antifungal therapy. Per the ESCMID/ECMM joint guideline, posaconazole and/or itraconazole are considered optimal therapy based on prior case reports. Similar to recent cases in the literature [17], our patient did not respond to initial presumptive treatment with isavuconazole. Whether this is due to a higher MIC is unclear. For example, a prior case that described the use of antifungal therapy based on susceptibilities for disseminated *Verruconis gallopava* in a renal transplant recipient showed that it was ultimately unsuccessful [10]. Nevertheless, this treatment failure adds to an increasing body of literature [8,10] that suggests unexpectedly high breakthrough fungal infection rates in immunocompromised patients on isavuconazole therapy. While the aforementioned meta-analysis only included hematologic malignancy and stem cell therapy patients, these data suggest that similar breakthrough infections could also occur in solid organ transplant patients.

To our knowledge, this is the first case of disseminated *Verruconis gallopava* in a solid organ transplant recipient receiving belatacept [18]. For our patient, clinical improvement and success subsequently occurred with amphotericin B and posaconazole, which included resolution of CNS infection, followed by continued improvement on posaconazole monotherapy. Success with posaconazole has also been seen in recent phaeohyphomycosis literature, including disseminated and CNS manifestations [19]. Future multicenter trials should move towards the development of standardized treatment guidelines, with particular regard to MIC interpretation, antifungal choice and duration, and utility of combination antifungal therapy.

## CRediT authorship contribution statement

**Matthew Cappiello:** Conceptualization, Investigation, Visualization, Writing – original draft. **Matthew Carlin Corpuz Bonus:** Conceptualization, Investigation, Validation, Writing – review & editing. **Sandy Chang:** Conceptualization, Investigation, Supervision, Visualization, Writing – review & editing. **Jonathan Arcobello:** Conceptualization, Investigation, Supervision, Visualization, Writing – review & editing.

## Conflict of interest

There are none.
